# Supplementary material for: The genes controlling normal function of citrate and spermine secretion are lost in aggressive prostate cancer and prostate model systems
Source: iScience. 2022 May 23;25(6):104451. doi: 10.1016/j.isci.2022.104451 (PMC9189124; doi:10.1016/j.isci.2022.104451)
Supplement: Document S1. Figures S1–S18 and Tables S1–S4 [file mmc1.pdf]

iScience, Volume *25*

Supplemental information

The genes controlling normal function of citrate and spermine secretion are lost in aggressive prostate cancer and prostate model systems

Morten Beck Rye, Sebastian Krossa, Martina Hall, Casper van Mourik, Tone F. Bathen, Finn Drabløs, May-Britt Tessem, and Helena Bertilsson

Supplemental Figures and Tables

Figure S1: Integrity of initial and refined citrate – spermine (CS) gene signatures, related to Figure 2.

CMS for the initial CS module based on the 40 normal samples from *Bertilsson.* (Dataset ID 1). **A)** The initial CMS was not significant in the *Bertilsson* (p=0.14, lognorm test), even though the normal samples CMS (0.37) was slightly higher compared to the CMS based on cancer samples (0.34). **B)** The initial CS module (blue dots) could not be validated in prostate cancer (red circles) and normal (blue circles) samples in 11 additional datasets. The CMS was not statistically significant in any of the datasets (lognorm test, Supplementary Table 3 and 4). Dataset ID 4, 5 and 8 did not include normal samples.

A)

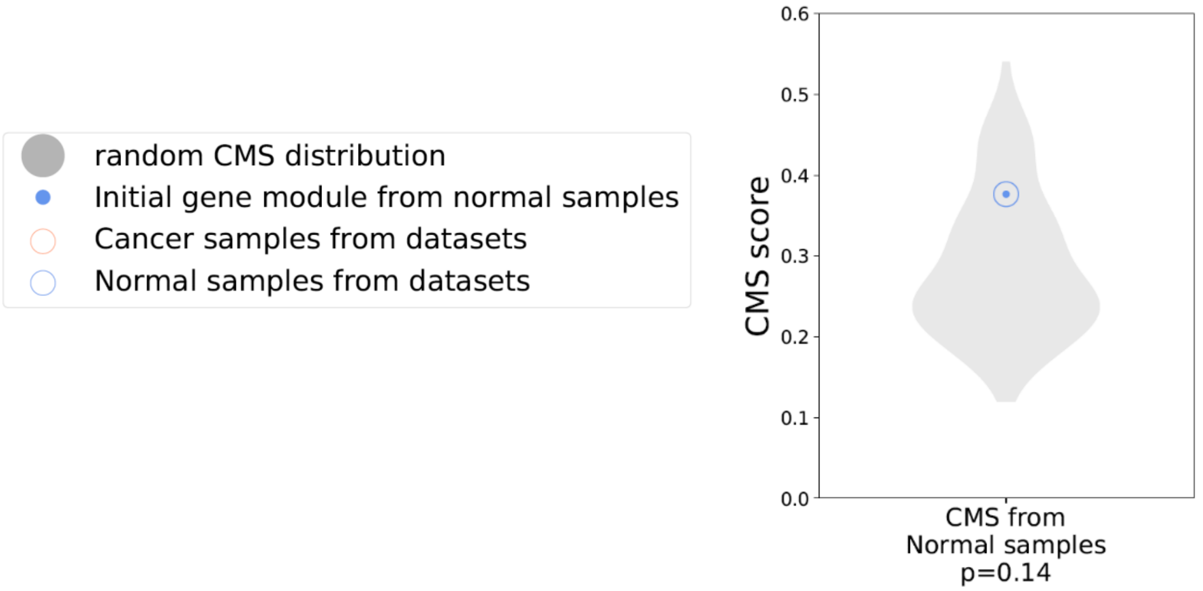

B)

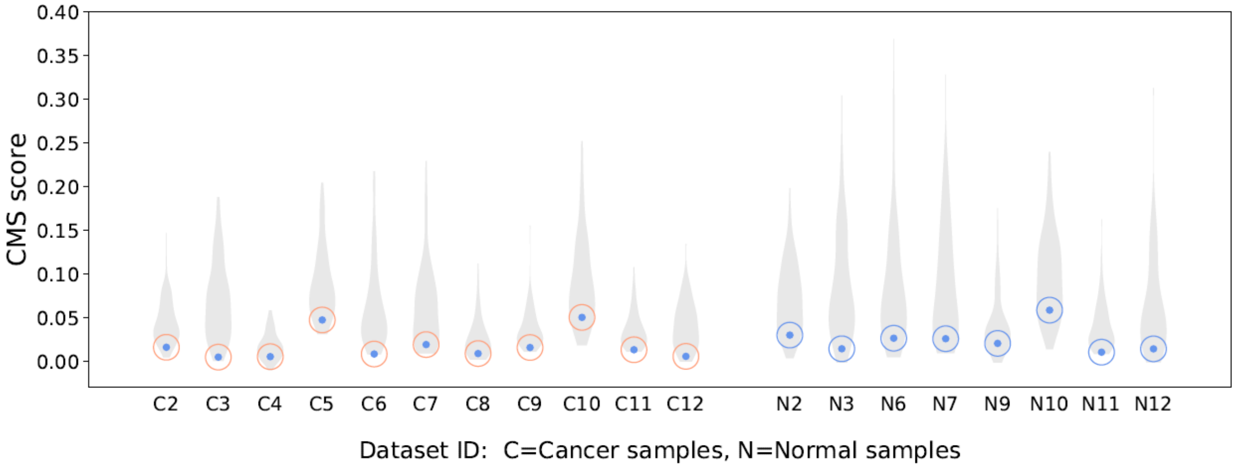

Table S1: CMS for the initial **cancer** sample CS gene module from *Bertilsson* evaluated on cancer samples in 12 datasets, related to Figure 2.

Dataset ID	Dataset Abbreviation	Cancer Samples	Number of genes	CMS score	Random average CMS score	p-value	Lognorm dist. lack-of-fit pvalue
1	Bertilsson	116	150	0.35	0.2	0.008	0.9
2	Chen	65	115	0.23	0.05	0.002	0.4
3	Taylor	131	143	0.24	0.07	0.03	0.4
4	Sboner	281	75	0.13	0.02	0.02	0.3
5	Erho	545	142	0.21	0.1	0.04	0.3
6	TCGA	497	147	0.23	0.06	0.007	0.7
7	CMBR	112	150	0.21	0.06	0.02	0.3
8	STCK	94	150	0.25	0.04	0.002	0.4
9	Mortensen	36	143	0.20	0.04	0.001	0.3
10	Prensner	78	147	0.21	0.09	0.04	1.0
11	Stopsack	264	144	0.16	0.04	0.0008	0.9
12	Kuner	59	140	0.17	0.05	0.02	0.1

Table S2: CMS for the initial **cancer** sample CS gene module from *Bertilsson* evaluated on normal samples in 9 datasets, related to Figure 2.

The data from *Mortensen* are from laser dissected tissue, and the lack of significant CMS is likely due to the more homogenous epithelial tissue samples, resulting in only subtle variations in CS genes

Dataset ID	Dataset Abbreviation	Normal Samples	Number of genes	CMS score	Random average CMS score	p-value	Lognorm dist. lack-of-fit pvalue
1	Bertilsson	40	150	0.35	0.09	0.01	0.9
2	Chen	71	115	0.25	0.05	0.01	0.2
3	Taylor	29	143	0.29	0.07	0.04	0.6
6	TCGA	52	147	0.27	0.05	0.006	0.3
7	CMBR	74	150	0.26	0.07	0.02	0.4
9	Mortensen	14	143	0.07	0.03	0.1	0.1
10	Prensner	38	144	0.23	0.07	0.02	1.0
11	Stopsack	160	144	0.17	0.03	0.0004	0.8
12	Kuner	39	140	0.22	0.04	0.004	0.5

Table S3: CMS for the initial **normal** sample CS gene module from *Bertilsson* evaluated on cancer samples in 12 datasets, related to Figure 2.

Dataset ID	Dataset Abbreviation	Cancer Samples	Number of genes	CMS score	Random average CMS score	p-value	Lognorm dist. lack-of-fit pvalue
1	Bertilsson	116	150	0.08	0.08	0.4	0.5
2	Chen	65	86	0.02	0.04	0.9	0.8
3	Taylor	131	127	0.00	0.07	1.0	0.1
4	Sboner	281	35	0.01	0.02	0.8	0.1
5	Erho	545	128	0.05	0.09	0.9	0.8
6	TCGA	497	140	0.01	0.06	1.0	0.8
7	CMBR	112	149	0.02	0.06	0.9	0.1
8	STCK	94	149	0.01	0.03	0.8	0.6
9	Mortensen	36	132	0.02	0.04	0.9	0.8
10	Prensner	78	147	0.05	0.09	0.8	0.8
11	Stopsack	264	139	0.01	0.04	0.9	1.0
12	Kuner	59	132	0.01	0.04	1.0	0.1

Table S4: CMS for the initial **normal** sample CS gene module from *Bertilsson* evaluated on normal samples in 9 datasets, related to Figure 2.

Dataset ID	Dataset Abbreviation	Normal Samples	Number of genes	CMS score	Random average CMS score	p-value	Lognorm dist. lack-of-fit pvalue
1	Bertilsson	40	150	0.34	0.27	0.2	0.4
2	Chen	71	86	0.03	0.07	0.9	0.1
3	Taylor	29	127	0.01	0.08	1.0	0.7
6	TCGA	52	140	0.03	0.09	0.9	0.9
7	CMBR	74	149	0.03	0.09	0.9	0.4
9	Mortensen	14	131	0.02	0.04	0.7	0.9
10	Prensner	38	144	0.06	0.10	0.8	0.5
11	Stopsack	160	139	0.01	0.04	1.0	0.4
12	Kuner	39	132	0.01	0.06	0.8	0.1

Figure S2: Validation of citrate-spermine gene signature, related to Figure 3

CS signature ssGSEA scores on spatial transcriptomics data from *Berglund* (dataset ID 20). The corresponding 12 pathological tissue images can be found in *Supplementary Information – Supplementary Figure 1b* in Berglund et al., 2018.

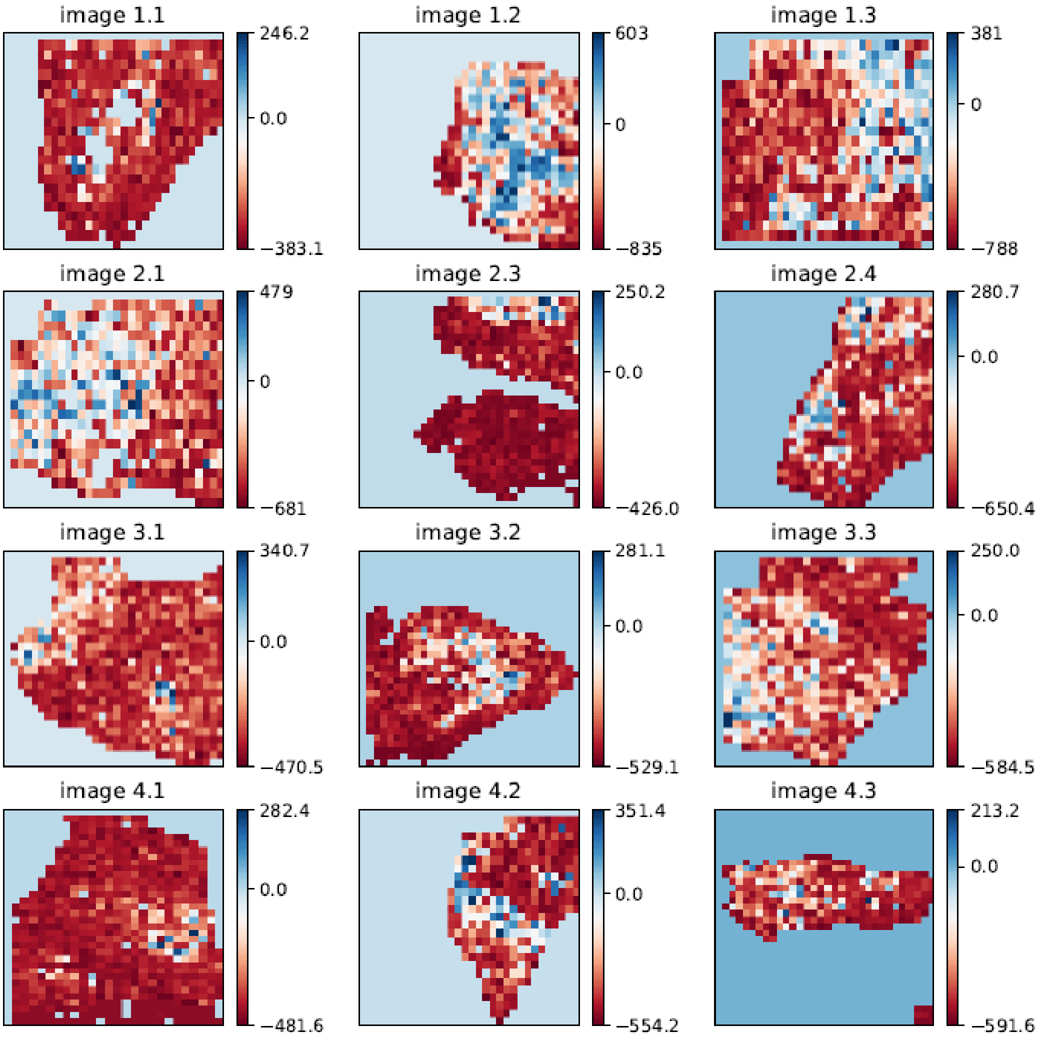

Figure S3: High stroma content confounds citrate-spermine signature ssGSEA scores in normal samples, related to Figure 4.

Stroma signature ssGSEA scores for prostate metastatis, cancer and normal prostate samples in 7 datasets. The scores were centered and normalized to range 0-1 before plotting to visualize similarities between datasets better. Normal prostate samples generally contain higher amount of stroma than cancer samples, which confounds differential expression analysis of CS signature scores involving normal prostate samples.

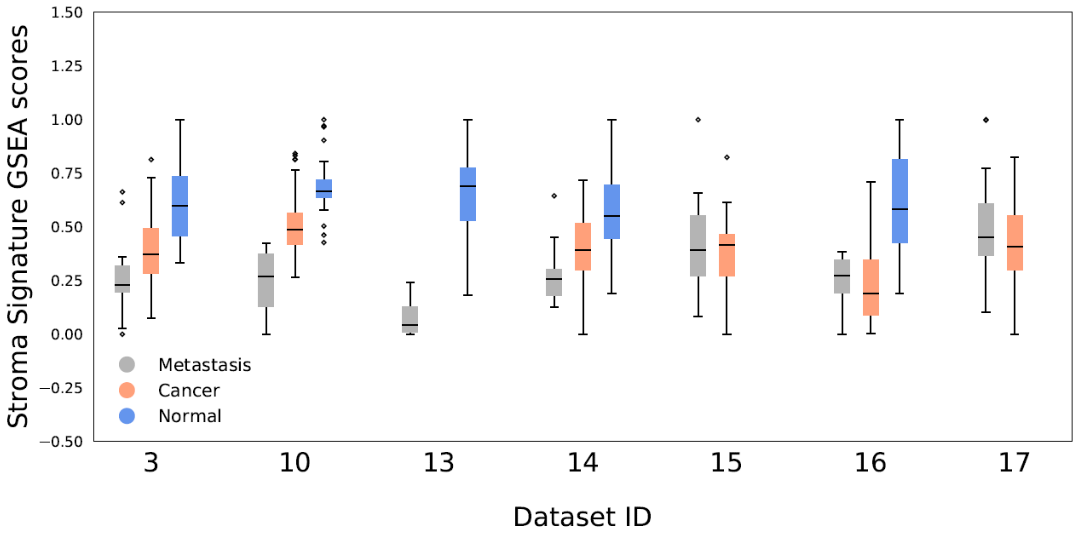

Figure S4: DAVID - Significant Gene Ontology Terms for the 150 genes in citrate-spermine gene signature, related to Figure 5.

Category	Term		Count	P_Value	Benjamini
**Annotation Cluster 1**	**Enrichment Score: 6.64**		**Count**	**P_Value**	**Benjamini**
UP_SEQ_FEATURE	region of interest:Alpha	RT	6	6.6E-9	2.8E-6
UP_SEQ_FEATURE	region of interest:Beta	RT	6	6.6E-9	2.8E-6
UP_SEQ_FEATURE	metal ion-binding site:Divalent metal cation; cluster B	RT	6	1.2E-8	2.5E-6
UP_SEQ_FEATURE	metal ion-binding site:Divalent metal cation; cluster A	RT	6	1.2E-8	2.5E-6
INTERPRO	Metallothionein, vertebrate, metal binding site	RT	6	1.9E-8	6.4E-6
SP_PIR_KEYWORDS	metal-thiolate cluster	RT	6	2.0E-8	5.5E-6
INTERPRO	Metallothionein superfamily, eukaryotic	RT	6	3.2E-8	5.4E-6
INTERPRO	Metallothionein, vertebrate	RT	6	3.2E-8	5.4E-6
PIR_SUPERFAMILY	PIRSF002564:metallothionein	RT	6	6.9E-8	6.1E-6
SP_PIR_KEYWORDS	cadmium	RT	5	2.4E-7	3.3E-5
SP_PIR_KEYWORDS	chelation	RT	5	2.4E-7	3.3E-5
SP_PIR_KEYWORDS	acetylated amino end	RT	9	1.1E-6	7.6E-5
GOTERM_MF_FAT	cadmium ion binding	RT	5	1.2E-6	4.6E-4
SP_PIR_KEYWORDS	metal binding	RT	5	1.2E-5	6.8E-4
GOTERM_MF_FAT	copper ion binding	RT	6	3.7E-4	2.8E-2
SP_PIR_KEYWORDS	copper	RT	5	9.9E-4	3.9E-2
**Annotation Cluster 2**	**Enrichment Score: 3.45**		**Count**	**P_Value**	**Benjamini**
GOTERM_MF_FAT	cofactor binding	RT	11	7.7E-5	1.4E-2
SP_PIR_KEYWORDS	nad	RT	9	1.1E-4	5.3E-3
GOTERM_MF_FAT	coenzyme binding	RT	9	2.2E-4	2.7E-2
GOTERM_MF_FAT	NAD or NADH binding	RT	4	8.5E-3	3.7E-1
**Annotation Cluster 3**	**Enrichment Score: 3.13**		**Count**	**P_Value**	**Benjamini**
KEGG_PATHWAY	Valine, leucine and isoleucine degradation	RT	8	5.4E-7	3.9E-5
KEGG_PATHWAY	Fatty acid metabolism	RT	7	5.3E-6	1.9E-4
KEGG_PATHWAY	Propanoate metabolism	RT	5	4.6E-4	1.1E-2
KEGG_PATHWAY	beta-Alanine metabolism	RT	4	2.0E-3	3.5E-2
KEGG_PATHWAY	Butanoate metabolism	RT	4	6.9E-3	7.9E-2
KEGG_PATHWAY	Tryptophan metabolism	RT	3	7.8E-2	4.1E-1
KEGG_PATHWAY	Lysine degradation	RT	3	9.2E-2	4.4E-1
**Annotation Cluster 4**	**Enrichment Score: 2.99**		**Count**	**P_Value**	**Benjamini**
GOTERM_BP_FAT	carboxylic acid catabolic process	RT	8	3.5E-5	1.9E-2

Figure S5: Enrichr - Pathways for the 150 genes in citrate-spermine gene signature, related to Figure 5

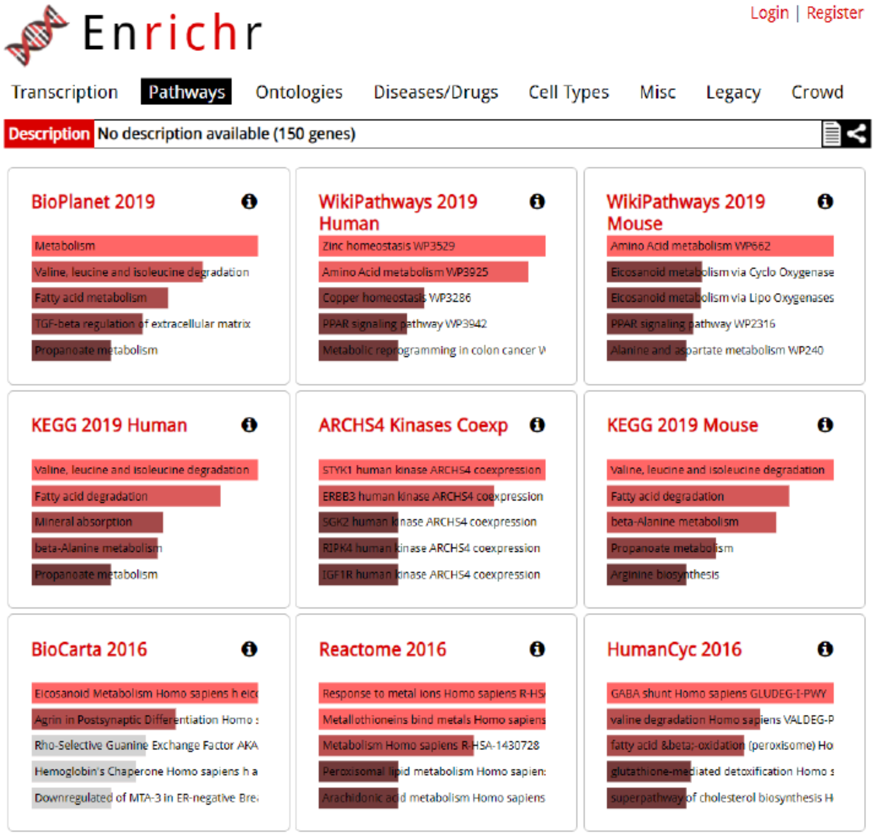

Enrichr - Ontologies

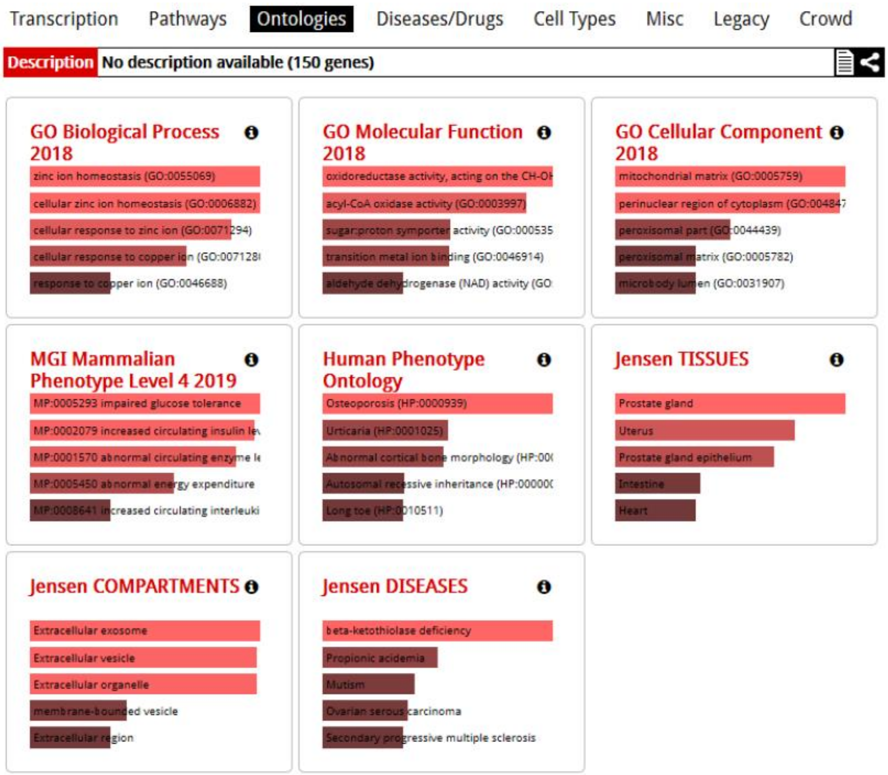

Figure S6: Network analysis of the 150 genes in the CS gene signature, related to Figure 5.

The network is a consensus over datasets 1-12.

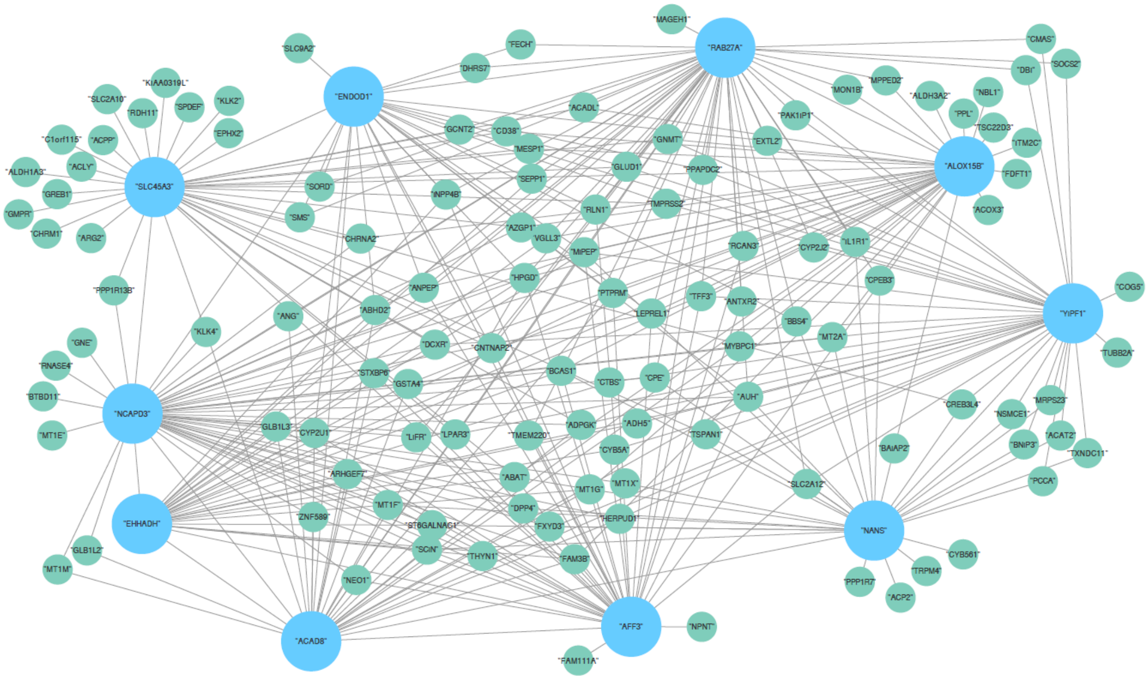

Figure S7: CS signature ssGSEA scores for all tissue types in *Taylor* (dataset ID 3), related to Figure 6.

This includes the four prostate cancer cell-types DU145, PC3, LNCaP and VCaP (one sample reach). The ssGSEA scores for cell-lines relative to other tissue types is similar to the *Prensner* dataset. Androgen responsive LNCaP and VCaP cell-types score higher than androgen resistant DU145 and PC3.

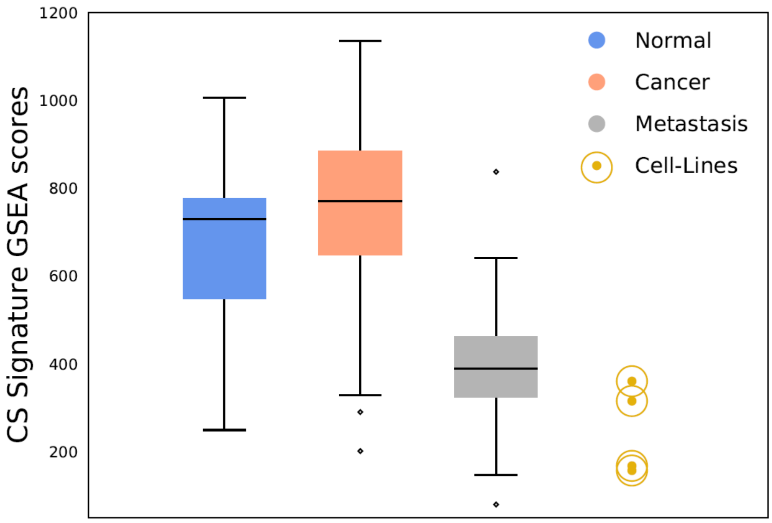

Figure S8: CS Signature ssGSEA scores for the three prostate cancer cell-types LNCaP, PC3 and DU145 in *Søgaard* (dataset ID 26), related to Figure 6.

There are 12 samples for each cell-type. The ssGSEA scores are highly reproducible within each cell-type, and the relative difference between the cell-types are similar to the differences observed in *Prensner* and *Taylor*, demonstrating that the assessment of CS signature enrichment of cell-lines is robust across cohorts.

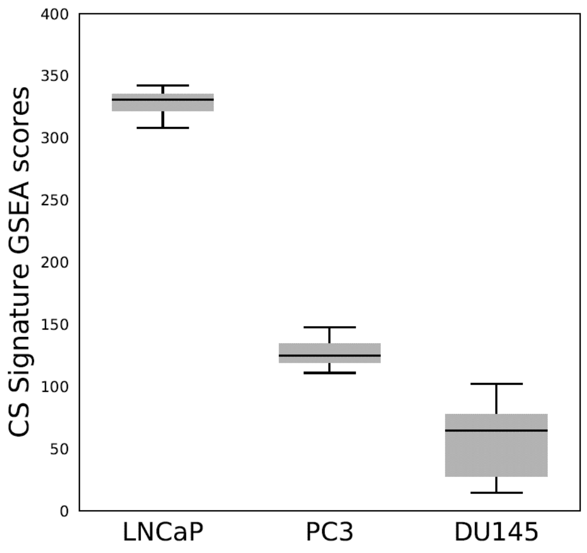

Figure S9: CS signature ssGSEA scores in 622 cancer cell-lines from *E-MTAB-2706* (dataset ID 25), related to Figure 6.

Androgen responsive cell-line LNCaP have higher ssGSEA score than androgen resistant cell-lines DU145 and PC3, which score similar to cell-types from other cancers. The results are similar to those observed in the *CCLE* dataset.

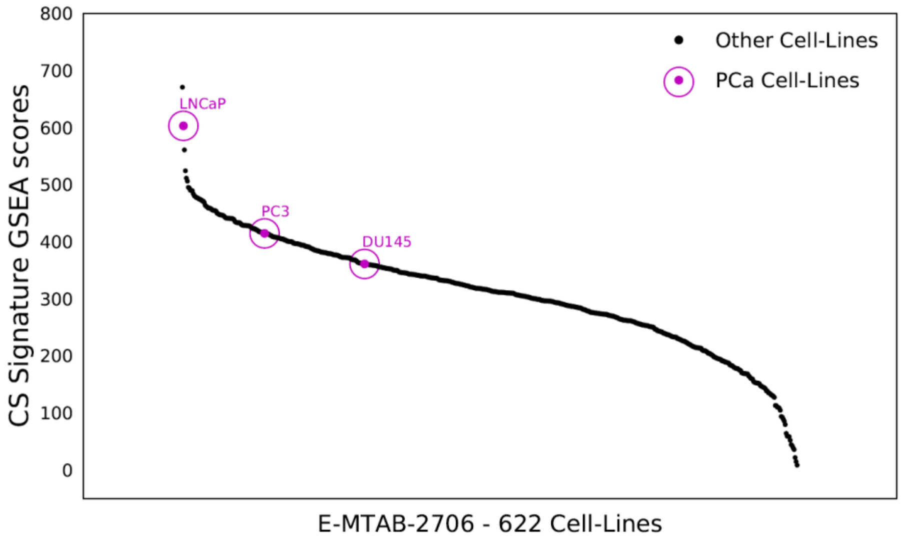

Figure S10: Adapted CS signature ssGSEA scores between the *Prensner* and *Taylor* datasets, related to Figure 6.

The ssGSEA scores shows that the adaptation procedure enable comparison of ssGSEA scores between different datasets. The *Taylor* dataset contains one sample from each of the cell-types DU145, PC3, LNCaP and VCaP.

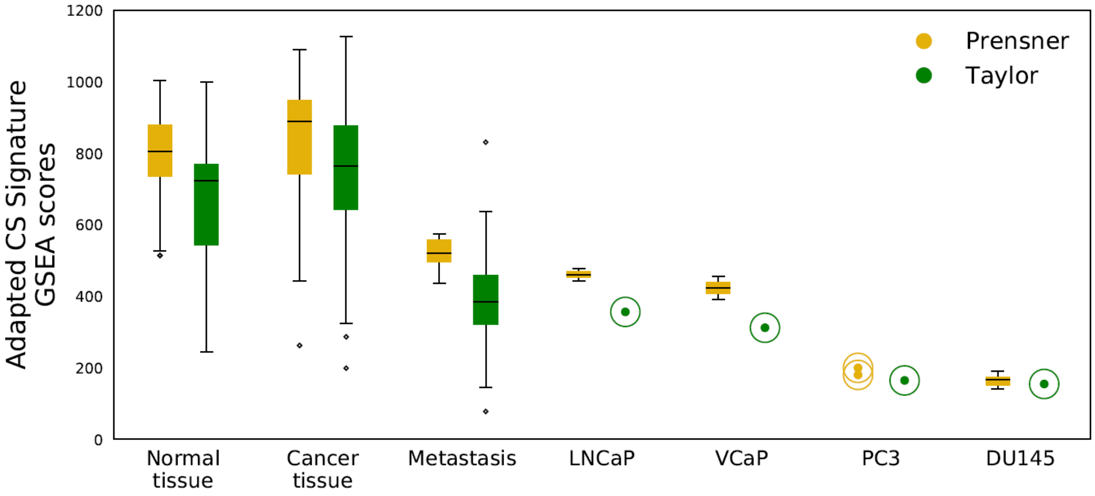

Figure S11 related to Figure 7, Figure 4D and Discussion (Signature Refinement): CS signature ssGSEA scores from 96 metastatic samples (9 from prostate) in the *Hsu* dataset using the initial CS signature before refinement. Without refinement, the CS signature is not able to separate all prostate metastatic samples from other metastatic samples.

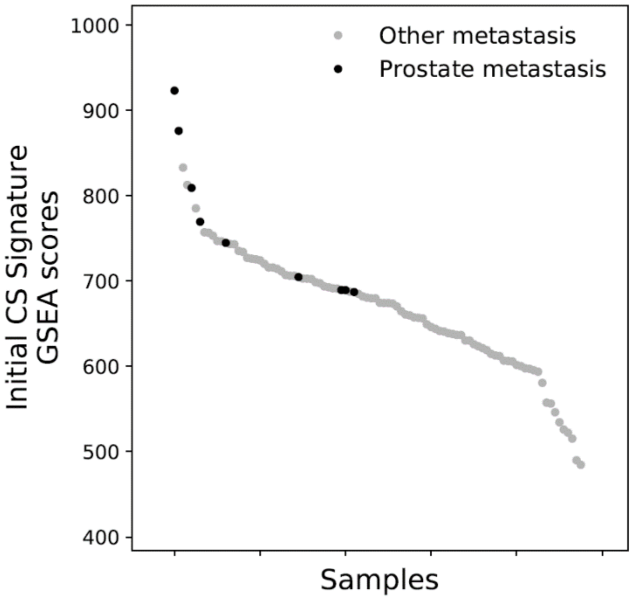

Figure S12 related to Figure 7, Figure 3C and Discussion (Signature Refinement): CS signature ssGSEA scores from the *FANTOM* dataset using the initial CS signature before refinement. Without refinement, the single Prostate Adult Tissue sample do not obtain the highest ssGSEA score.

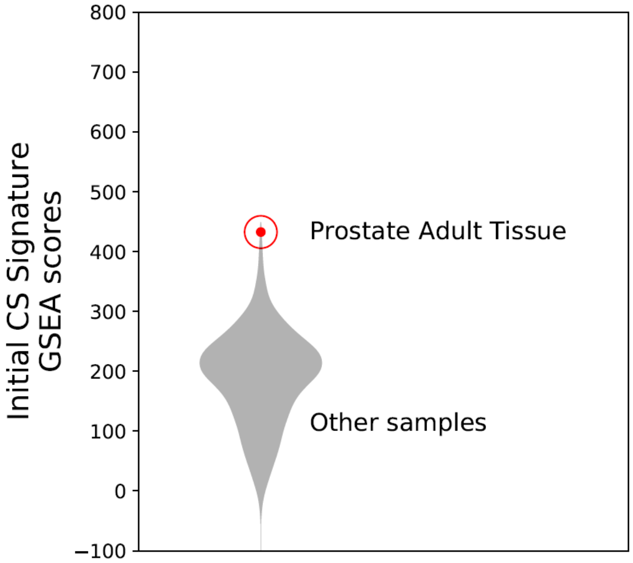

Figure S13 related to Figure 7, Figure 3A and Discussion (Signature Refinement): Improvement of CS ssGSEA scores after refinement for cancer and normal samples in the *TCGA-complete* dataset. The ssGSEA score improvements are more pronounced in prostate cancer (*PRAD*) compared to other cancers. This shows that the refinement procedure for the CS signature is most effective for prostate. In addition, the results show that increased ssGSEA scores after refinement depend on the tissue type, and is not a general bias from the refinement procedure.

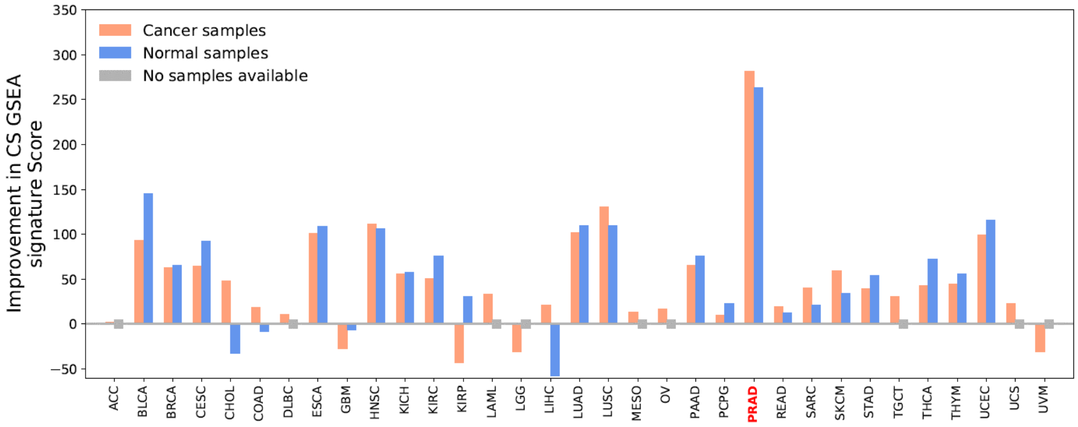

Figure S14 related to Figure 7 and Discussion (Signature Refinement): Refined gene signature average GSEA scores for 37 metabolite and lipid-signal signatures evaluated for all 33 cancers in the *TCGA-complete* dataset. Of all metabolites and lipids, citrate and spermine have the most elevated prostate cancer GSEA scores relative to other cancers. This shows, 1) That different gene signatures with different properties are produced for different metabolites, and 2) That gene signatures for citrate are spermine are created specifically in the prostate TCGA samples, and not samples from other TCGA cancers.

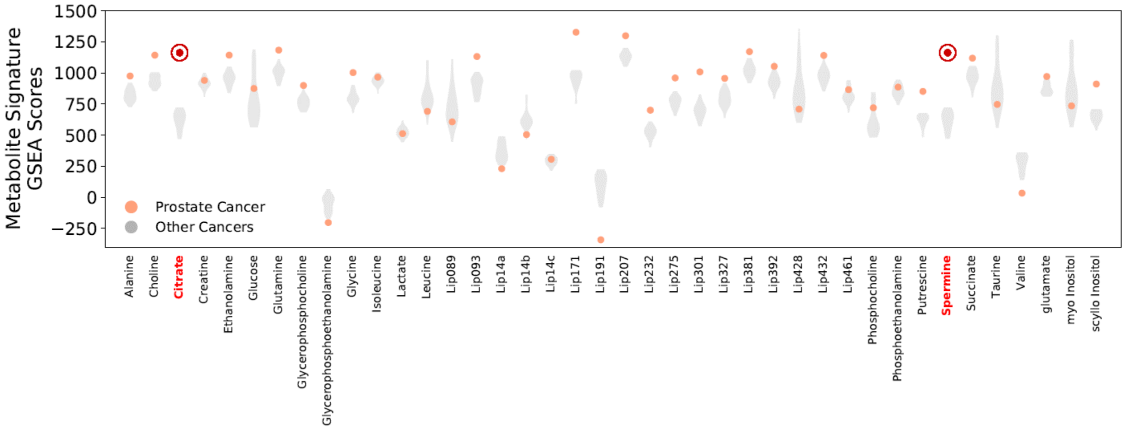

Figure S15 related to Figure 7 and Discussion (Signature Refinement): Refined gene signature average GSEA scores for 37 metabolite and lipid-signal signatures evaluated for all 33 cancer in the *TCGA-complete* dataset. The figure is based on Supplementary Figure 13, but now the average GSEA score for other cancers have been subtracted from the prostate cancer GSEA score to emphasize the metobolite and lipid signals with the largest difference. This figure highlights whether a particular metabolite is regulated by correlated genes unique for prostate.

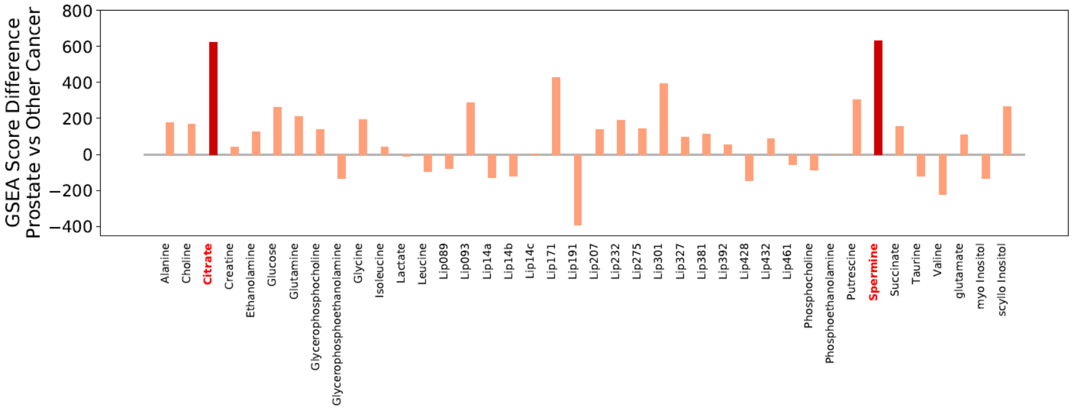

Figure S16 related to Figure 7 and Discussion (Signature Refinement): Same as Supplementary Figure 13, but now for normal samples for 24 tissues in the *TCGA-complete* dataset. The results are the same as for the cancer samples.

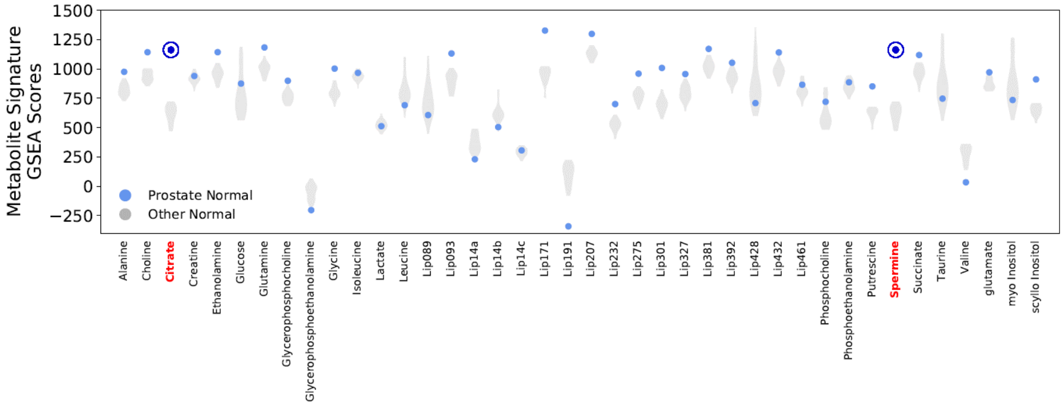

Figure S17 related to Figure 7 and Discussion (Signature Refinement): Same as Supplementary Figure 14, but now for normal samples for 24 tissues in the *TCGA-complete* dataset. The results are the same as for the cancer samples.

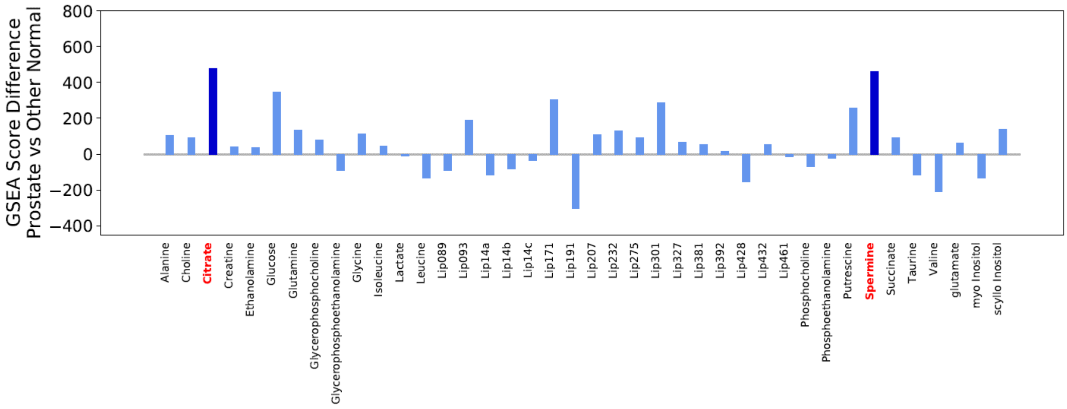

Figure S18 related to Figure 7, Figure 3C and Discussion (Signature Refinement): Refined Metabolite signature GSEA score ranks for the one Prostate Adult Tissue samples in the *FANTOM* dataset (1829 samples in total) after signature refinement. Four metabolites recieved the highest rank (rank 0) for prostate adult tissue; citrate, spermine, putrescine, and the lipid signal Lip301. The ranks before signature refinement for these signals where 3, 4, 16 and 11 respectively, demonstrating that refinement improved the prostate specificity of the CS gene signature. A high rank would indicate that metabolites (or lipids) are differently regulated in prostate tissue compared to other tissues and cell-lines.

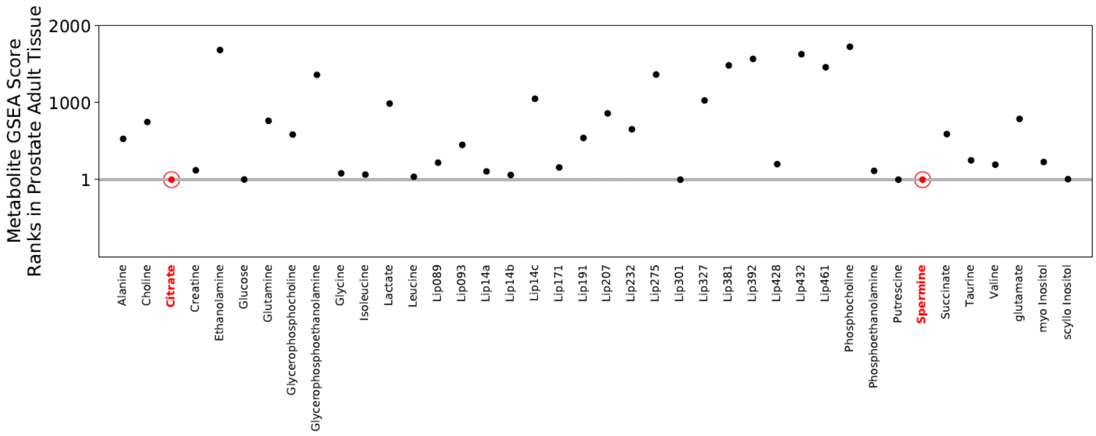

Supplemental Figures and Tables end.
